# Supplementary material for: Variable Selection for Multivariate Failure Time Data via Regularized Sparse-Input Neural Network
Source: Bioengineering (Basel). 2025 May 31;12(6):596. doi: 10.3390/bioengineering12060596 (PMC12189315; doi:10.3390/bioengineering12060596)
Supplement: Supplementary file 1 [file bioengineering-12-00596-s001.zip › bioengineering-3648472-supplementary.pdf]

A supplementary file for "Variable Selection for Multivariate Failure Time Data via Regularized Sparse-Input Neural Network"

Bin Luo [1] and Susan Halabi [*2]

[1]School of Data Science and Analytics, Kennesaw State University, GA, USA

[2]Department of Biostatistics and Bioinformatics, Duke University, NC, USA

A Implementation Details

A.1 Simulation studies

We employed Random Forest (RF) with 1000 decision trees for the model fitting process. To ensure a fair comparison among all the neural-net-based methods, we adopted a ReLu-activated Multi-Layer Perceptron (MLP) with two hidden layers consisting of 10 and 10 units, respectively. Network weights were initialized from a Gaussian distribution with mean zero and standard deviation 0.1, while bias terms were set to zero, to ensure a consistent starting point for optimization and to help break symmetry in the network. The gradient descends step in Algorithm 1 is implemented by Adam optimizer with an initial learning rate of 0.001. Since Adam generates an adaptive learning rate for each update, the learning rate in the proximal operator is challenging to track. Therefore, we treat γ as a hyperparameter. Our empirical results indicate that setting $\gamma = 1$ yields consistently robust performance across various scenarios. Hence, this is used throughout our computations, reflecting its effectiveness and stability.

The parameter search ranges for λ and α are displayed in Table S.1. These ranges are chosen to generate a solution path from a dense model, where most of the variables are selected, to a null model, where none of the variables are included. For all the methods

*Corresponding author. Email: susan.halabi@duke.edu

falling within the framework of Equation (1) in the paper, we selected the optimal values of λ and α from a two-dimensional grid, with λ and α ranging over 50 and 10 evenly spaced values on a logarithmic scale, respectively. The selection was based on their performance on the validation set, which consisted of 20% of the training set. To deactivate feature selection, we set $\lambda = 0$ for Oracle-NN. For the regularized methods with variable selection, the number of epochs at λ_{min} was set to 2000 for the low-dimensional (LD) scenario and 200 for the high-dimensional (HD) scenario. For all other values of λ, the number of epochs was set to 200 for both LD and HD settings. The number of epochs for oracle-NN was consistently fixed at 5000.

Table S1: **List of the search range for the tuning parameters used in our simulation.**

<table>
<tr><td rowspan="3">Method</td><td colspan="4">Search Range</td></tr>
<tr><td colspan="2">LD</td><td colspan="2">HD</td></tr>
<tr><td>λ</td><td>α</td><td>λ</td><td>α</td></tr>
<tr><td>LASSO</td><td rowspan="3">[0.0005, 0.002]</td><td rowspan="7">[0.001, 0.1]</td><td rowspan="3">[0.0005, 0.002]</td><td rowspan="7">[0.01, 0.1]</td></tr>
<tr><td>Bi-LASSO</td></tr>
<tr><td>Bi-GLASSONet</td></tr>
<tr><td>MCP</td><td rowspan="4">[0.001, 0.5]</td><td rowspan="4">[0.01,0.2]</td></tr>
<tr><td>GMCPNet</td></tr>
<tr><td>Bi-MCP</td></tr>
<tr><td>Bi-GMCPNet</td></tr>
</table>

A.2 Real Data Example

For the bivariate survival analysis on the CALGB-90401 dataset, the implementation details remain the same as in the high-dimensional (HD) scenario in the simulation studies, with the following modifications: In hyperparameter tuning, we explored 100 values of λ ranging from 0.01 to 0.1 for MCP and GMCPNet, and from 0.01 to 0.05 for Bi-GMCPNet. Additionally, we increased the number of candidates for α to 50. The CALGB 90401 data is available from the NCTN Data Archive at `ttps://nctn-data-arcive`.nci.nih.gov/.

B Additional Simulation Results

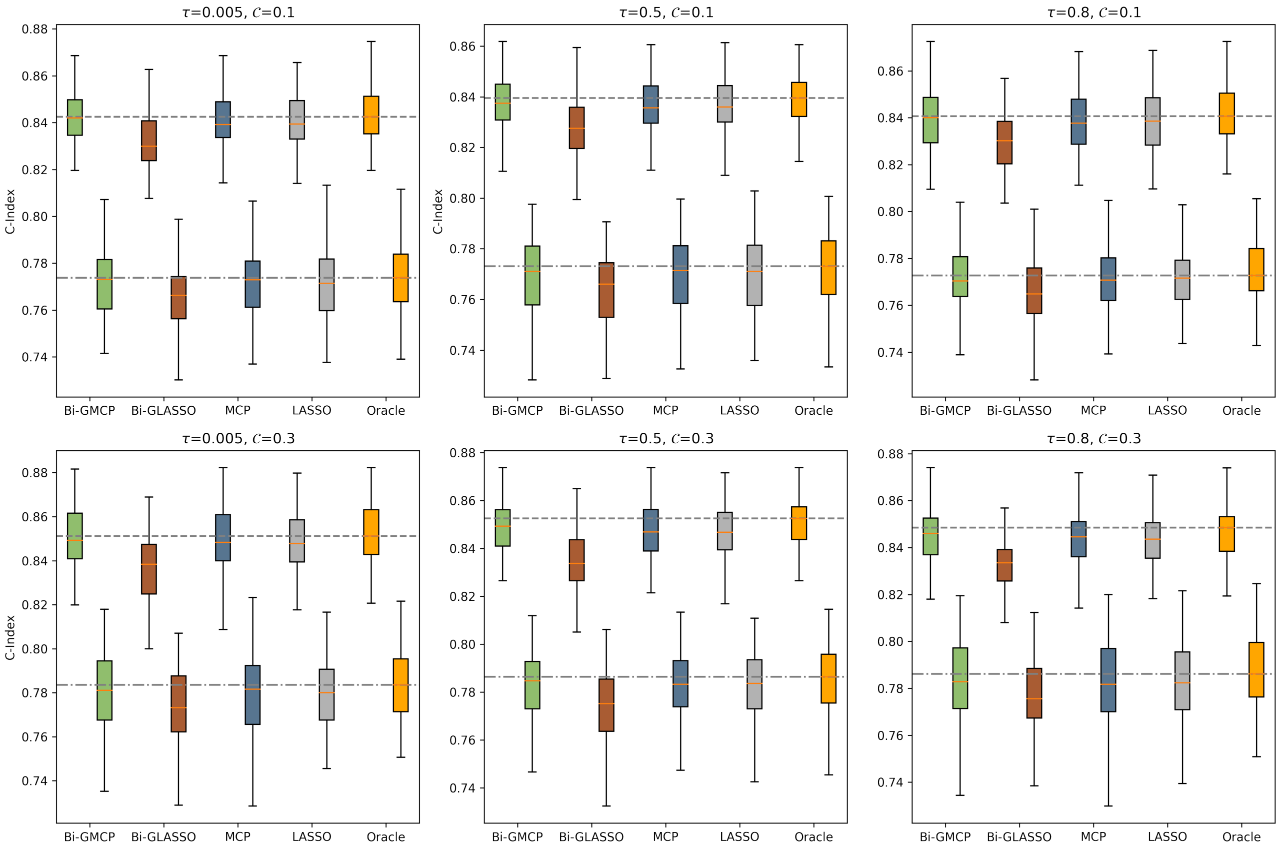

Figure S1: **C-Index comparison of the proposed methods within the linear bivariate failure time model (Example 1) for sample size $n = 300$, with censoring rate $= 0.1$ and 0.3, and Kendall's τ values $\tau = 0.005, 0.5$ and 0.8.** Each method is represented by two boxplots for each outcome. The dashed lines within each panel denote the median scores of the Oracle method, serving as a benchmark for comparison.

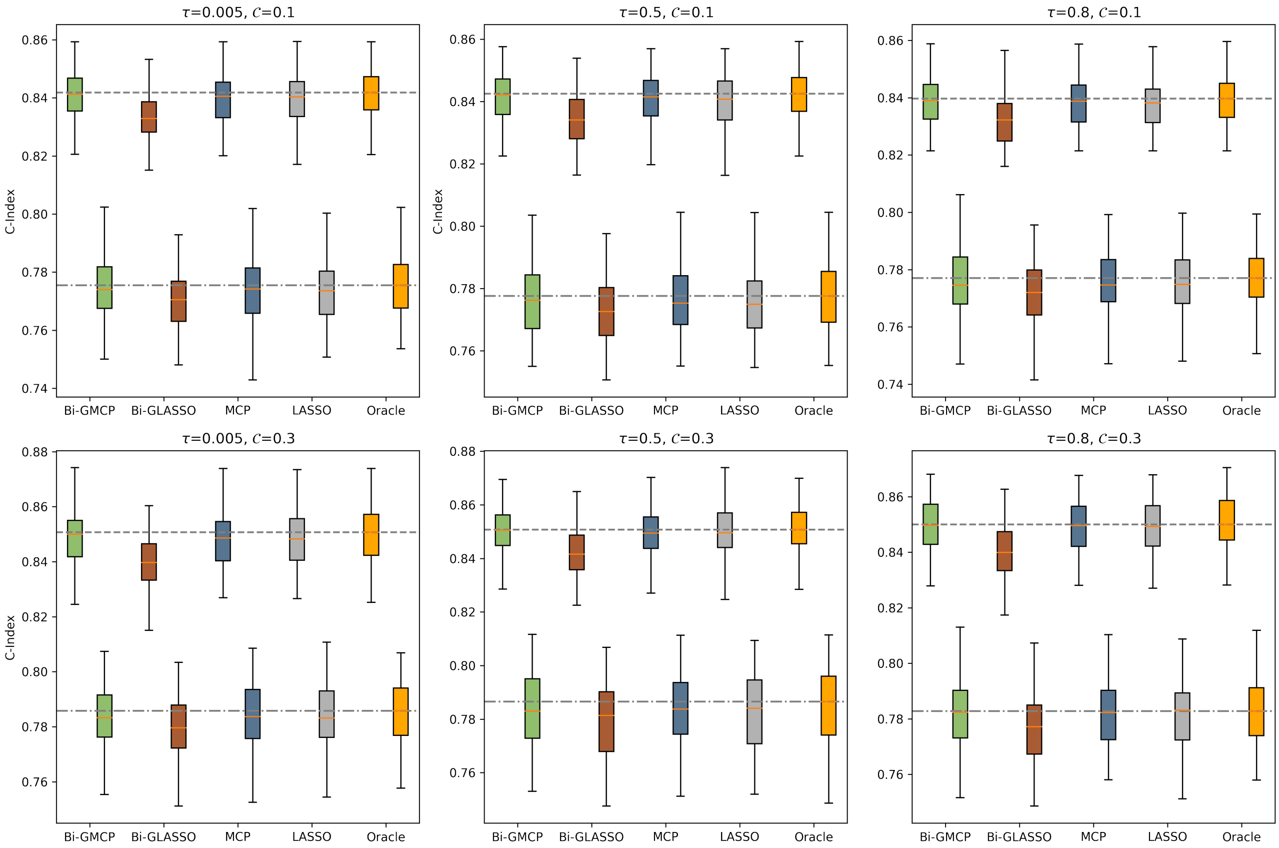

Figure S2: **C-Index comparison of the proposed methods within the linear bivariate failure time model (Example 1) for sample size $n = 500$, with censoring rate $= 0.1$ and 0.3, and Kendall's τ values $\tau = 0.005, 0.5$ and 0.8.** Each method is represented by two boxplots for each outcome. The dashed lines within each panel denote the median scores of the Oracle method, serving as a benchmark for comparison.

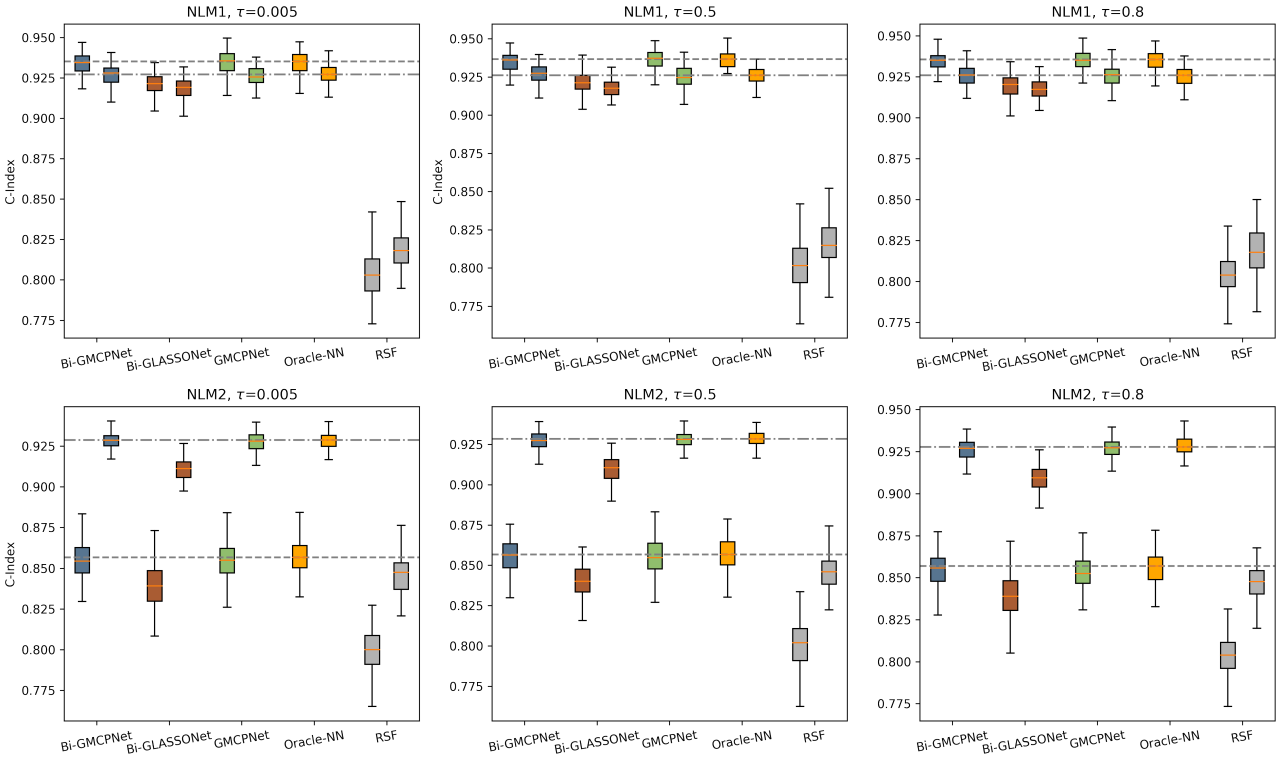

Figure S3: **C-Index comparison of proposed methods in the context of the non-linear bivariate failure time model (Example 2) with Kendall's** $\tau = 0.005, 0.5$ **and** 0.8. The figure comprises four panels, illustrating two models (NLM1 and NLM2) under low-dimensional setting $p = 20$. In each panel, methods are represented by two boxplots for each outcome, and dashed lines indicate the median scores of the Oracle-NN, serving as a benchmark for comparison.

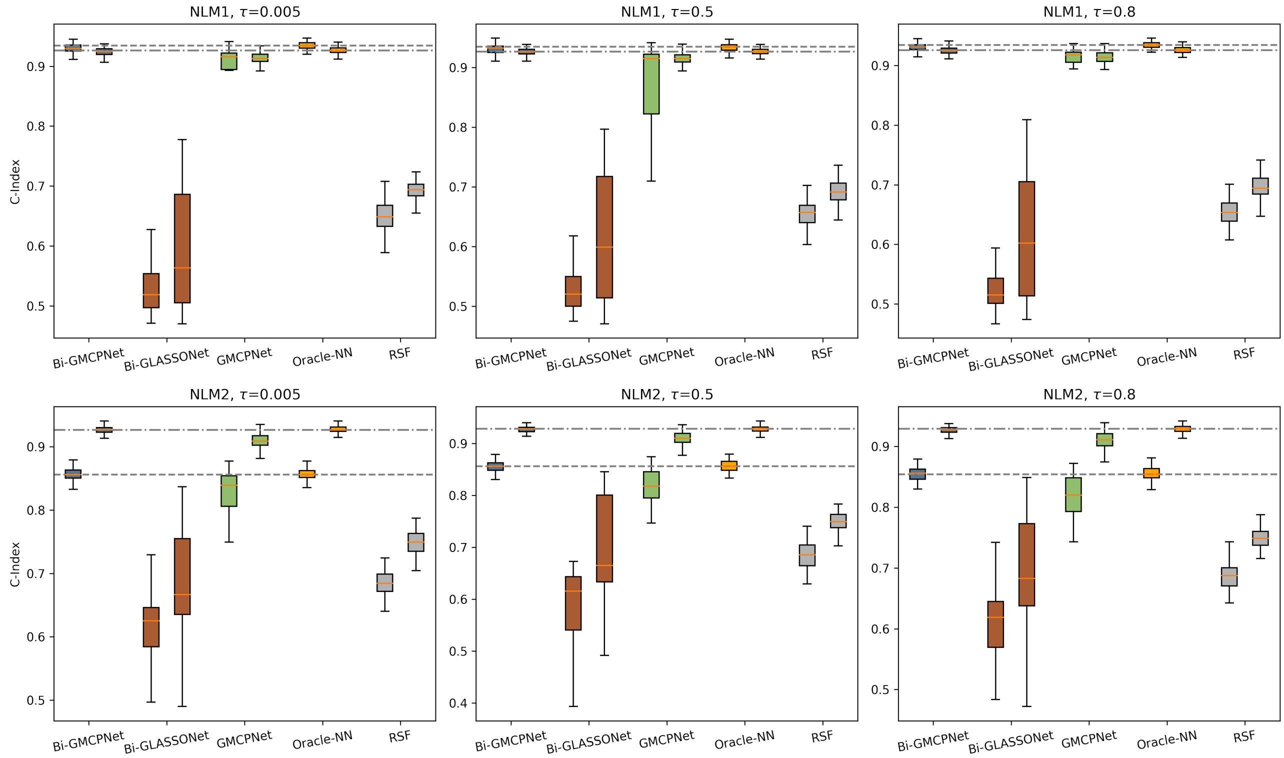

Figure S4: **C-Index comparison of proposed methods in the context of the non-linear bivariate failure time model (Example 2) with Kendall's** $\tau = 0.005, 0.5$ **and** 0.8. The figure comprises four panels, illustrating two models (NLM1 and NLM2) under high-dimensional setting $p = 1000$. In each panel, methods are represented by two boxplots for each outcome, and dashed lines indicate the median scores of the Oracle-NN, serving as a benchmark for comparison.
